# Idiopathic Sporadic Onychomadesis of Toenails

**DOI:** 10.1155/2016/6451327

**Published:** 2016-06-29

**Authors:** Poonkiat Suchonwanit, Sunatra Nitayavardhana

**Affiliations:** Division of Dermatology, Faculty of Medicine, Ramathibodi Hospital, Mahidol University, Bangkok 10400, Thailand

## Abstract

Onychomadesis is a clinical sign of nail plate separation due to transient or permanent arrest of nail matrix activities. Onychomadesis can be considered as a severe form of Beau's line. This condition usually occurs after trauma, causal diseases, or medications, yet it rarely occurs as an idiopathic condition. We report a case of a 38-year-old Thai female who developed recurrence onychomadesis in several toenails in the absence of predisposing factors or associated conditions. To the best of our knowledge, our patient is the first reported case of idiopathic onychomadesis limited to toenails.

## 1. Introduction

Onychomadesis is a clinical sign of nail plate separation due to transient or permanent arrest of nail matrix activities. In an early stage, the nail plate shows a transverse depression line (Beau's line). If the nail matrix remains injured for a long period of time, then the line becomes deeper until splitting of the nail plate; as a result, shedding of the nail occurs (onychomadesis). Onychomadesis can be considered as a severe form of Beau's line [1]. This condition usually occurs after trauma, causal diseases, or medications, yet it rarely occurs as an idiopathic condition. We report a case of a 38-year-old Thai female who developed recurrence onychomadesis in several toenails in the absence of predisposing factors or associated conditions.

## 2. Case Report

A 38-year-old Thai female presented with a 10-year history of asymptomatic shedding of several toenails. The symptom occurred in all toenails, usually in 1-2 nails at the same time. Nail shedding developed approximately 3-4 times per year from the proximal to the distal end, always returning to normal regrowth. Her fingernails were never affected. She had mild allergic rhinitis for more than 20 years which was well controlled. She reported taking oral antihistamines only for symptomatic treatment. She denied history of other systemic diseases or traumas prior to this condition. There were no family members having a similar problem. She preferred having manicure and pedicure with nail polish periodically.

**Figure 1:**
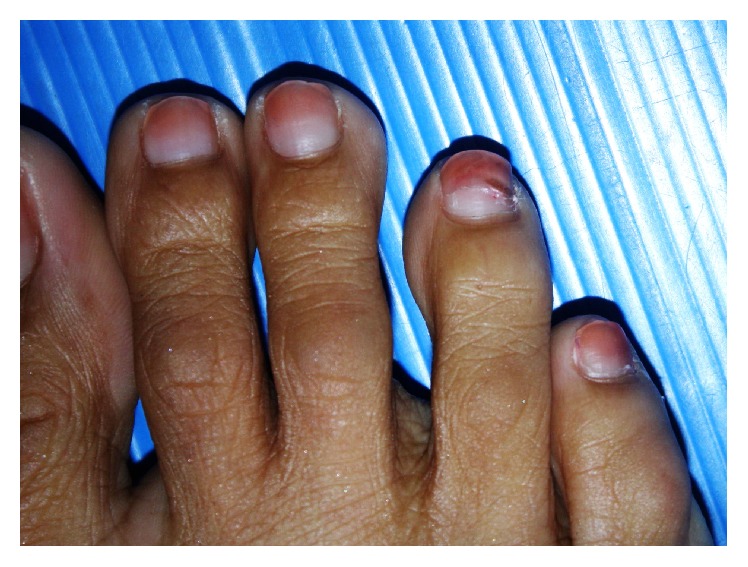
Proximal separation of the right 4th toenail.

**Figure 2:**
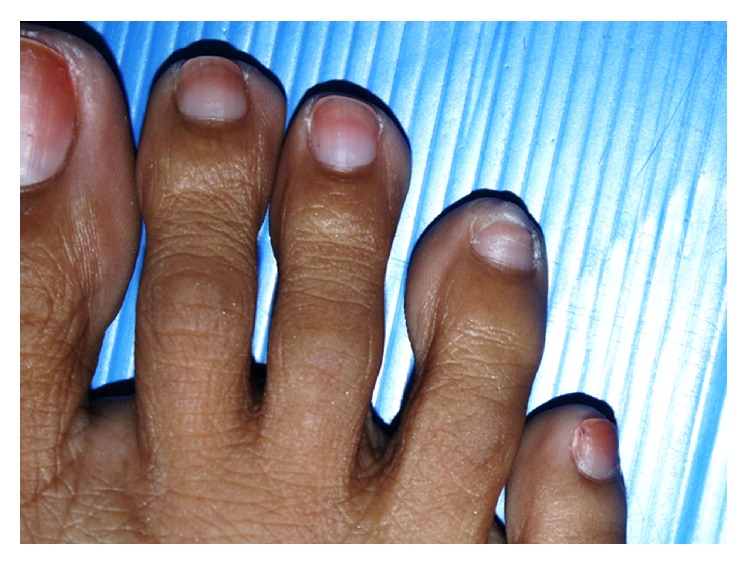
The affected toenail at the 4-month follow-up visit.

Physical examination revealed the proximal separation of the right 4th toenail from the nail bed and shallow transverse grooves on the right 3rd and 4th toenails (Figure 1). There was no subungual hyperkeratosis and there were no periungual lesions. The cuticle was intact. Fingernails and other toenails were normal. All nails were partially stained by unremoved nail polish. There was no evidence of other dermatological conditions. Complete blood count, transaminases, blood urea nitrogen, creatinine, thyroid function tests, antinuclear antibody test, and urinalysis revealed no abnormalities. The patient refused performing a nail matrix biopsy due to fear of pain. According to the patient's presentation, the diagnosis of idiopathic sporadic onychomadesis of toenails was performed. The patient was advised to practice gentle nail care and was asked to observe any events that may suggest the precipitating cause. The affected toenail improved at the 4-month follow-up visit (Figure 2).

## 3. Discussion

Onychomadesis is characterized by nail plate separation from the nail bed. The condition begins at the proximal part of the nail, possibly caused by the temporary or permanent arrest of the nail matrix cell activities. This condition is considered a similar pathophysiology to Beau's line, the transverse ridging of the nail plate, but it occurs in a higher degree of severity. The causes of onychomadesis can be idiopathic or associated with several conditions. The previous articles mostly reported that hand-foot-mouth disease, pemphigus vulgaris, and chemotherapeutic agents are the common associated conditions. Hardin and Haber have classified the associated conditions into autoimmune disease, other major medical illnesses, medication, neonatal illness, infection, and idiopathic cause [2].

Most of the reported cases associated the development of onychomadesis with the activity of systemic disease or using of a new drug. The clinical presentation is described as a temporary course with subsequent nail regrowth which affects several nails with the same degree of severity. On the other hand, onychomadesis occurring limited to single or a few nails suggests that the precipitating causes should directly affect the nail(s) including trauma or primary infection to the nail matrix. In our patient, she had a history of asymptomatic recurrent nail shedding in several toenails approximately 3-4 times per year and reported no association with health status, medications, or environmental factors. Nail changes randomly occurred, limited to 1-2 nails with spontaneous normal regrowth. The patient denied history of severe illness or trauma prior to this condition. Her presentation suggests the diagnosis of idiopathic onychomadesis.

The cases of idiopathic onychomadesis have been reported and can be classified as familial and sporadic. In 1897, Montgomery first reported a familial case of idiopathic onychomadesis [3]. Since then, there have been 3 reports of cases without underlying conditions presenting with periodic nails shedding and a family history, suggesting an autosomal dominant inheritance pattern [4–6]. Mehra et al. introduced the term idiopathic familial onychomadesis in 2000 for diagnosing this rare entity [6]. To our knowledge, there are 2 reports of nonfamilial idiopathic onychomadesis. The first case of idiopathic onychomadesis without a family history was reported by Venugopal and Murrell in 2009. They reported a 79-year-old Caucasian man who presented with seasonal fingernail shedding, usually in winter, for 10 years [7]. The other case was reported by Hardin and Haber in 2012. They described a 14-year-old Asian girl with a history of periodic shedding of her fingernails for 3 years and suggested the term idiopathic sporadic onychomadesis for the healthy patient with no family history and no seasonal nail shedding [8]. Our patient is the third reported case of idiopathic onychomadesis and fits the term idiopathic sporadic onychomadesis. Interestingly, idiopathic sporadic onychomadesis limited to toenails has never been reported before. Although the term “idiopathic” refers to unknown or uncertain cause, we hypothesize that microtrauma affecting those who have fragile nail matrix might play a role in the pathophysiology of idiopathic onychomadesis. The summary of previously reported cases is shown in Table 1.

The pathophysiology of onychomadesis is still unknown. Inhibition or arrest of the nail matrix cell activities would be one explanation. Another explanation is that the matrix cell activities remain normal, but the nail plate quality changes by becoming thinner and shedding [9]. The history of systemic condition or drug usage is considered. Generally, there is no specific treatment for onychomadesis. However, investigation and correction of the underlying conditions are the most important strategies. Our patient was advised on the general nail care and was asked to observe any events that may discover the precipitating cause in the future. The prognosis in most cases of onychomadesis is excellent. This condition usually temporarily occurs and is followed by complete normal looking nail regrowth.

In conclusion, we herein present an interesting case of idiopathic sporadic onychomadesis. To the best of our knowledge, our patient is the first reported case of idiopathic onychomadesis limited to toenails. However, it is important for clinicians to investigate the underlying conditions in patients with onychomadesis.

## Figures and Tables

**Table 1:** Clinical characteristics of previously reported cases of idiopathic onychomadesis.

Article	Age (Y)	Sex	Presenting symptom	Onset and duration	History of affected nail	Family history	Physical examination
Montgomery, 1897 [3]	35	Male	Shedding of nail plate	Infancy	Both	Yes	Right thumbnail loosened
Oliver, 1927 [4]	12	Male	Shedding of big toenail	2 years	Toenails	Yes	Right big toenail loosened
Martin and Rudolph, 1980 [5]	25	Male	Shedding of nail plate	Childhood	Both	Yes	All toenails loosened, with 7 affected fingernails
Mehra et al., 2000 [6]	12	Female	Asymptomatic nail shedding	4 months	Both	Yes	Separation of both 3rd fingernails, left 4th toenail, and right big toenail
Venugopal and Murrell, 2009 [7]	79	Male	Nail shedding every winter	10 years	Fingernails	No	Beau's line of all fingernails with toenail dystrophy
Hardin and Haber, 2012 [8]	14	Female	Periodic shedding of fingernails 2-3 times/year	3 years	Fingernails	No	Separation of left thumbnail with intact nail plate
Present case	38	Female	Periodic shedding of toenails 3-4 times/year	10 years	Toenails	No	Separation of right 4th toenail
